# Functional fibrinogen (FLEV-TEG) versus the Clauss method in an obstetric population: a comparative study

**DOI:** 10.1186/s12871-019-0769-8

**Published:** 2019-06-01

**Authors:** Alessandra Spasiano, Carola Matellon, Daniele Orso, Alessandro Brussa, Maria Cafagna, Anna Marangone, Teresa Dogareschi, Tiziana Bove, Roberta Giacomello, Desrè Fontana, Luigi Vetrugno, Giorgio Della Rocca

**Affiliations:** 1 Anesthesiology and Intensive Care Medicine, Department of Medicine, University of Udine, P.le S. Maria della Misericordia 15, 33100 Udine, Italy; 2 Department of Laboratory Medicine, ASUIUD Hospital of Udine, Udine, Italy; 3 Postgraduate School of Clinical Pathology and Biochemistry, University of Padua, Padua, Italy

**Keywords:** Thromboelastography, Post-partum hemorrhage, Coagulopathy, Fibrinogen

## Abstract

**Background:**

Hemostasis is the dynamic equilibrium between coagulation and fibrinolysis. During pregnancy, the balance shifts toward a hypercoagulative state; however placental abruption and abnormal placentations may lead to rapidly evolving coagulopathy characterized by the increased activation of procoagulant pathways. These processes can result in hypofibrinogenemia, with fibrinogen levels dropping to 2 g/L or less and an associated increased risk of post-partum hemorrhage.

The aim of the present study was to evaluate the concordance between two methods of functional fibrinogen measurement: the Thromboelastography (TEG) method (also known as FLEV) vs. the Clauss method. Three patient groups were considered: healthy volunteers; non-pathological pregnant patients; and pregnant patients who went on to develop postpartum hemorrhage.

**Methods:**

A prospective observational study. Inclusion criteria were: healthy volunteer women of childbearing age, non-pathological pregnant women at term, and pregnant hemorrhagic patients subjected to elective or urgent caesarean section (CS), with blood loss exceeding 1000 mL. Exclusion criteria were age < 18 years, a history of coagulopathy, and treatment with contraceptives, anticoagulants, or antiplatelet agents.

**Results:**

Bland-Altman plots showed a significant overestimation with the FLEV method in all three patient groups: bias was − 133.36 mg/dL for healthy volunteers (95% IC: − 257.84; − 8.88. Critical difference: 124.48); − 56.30 mg/dL for healthy pregnant patients (95% IC: − 225.53; 112.93. Critical difference: 169.23); and − 159.05 mg/dL for hemorrhagic pregnant patients (95% IC: − 333.24; 15.148. Critical difference: 174.19). Regression analyses detected a linear correlation between FLEV and Clauss for healthy volunteers, healthy pregnant patients, and hemorrhagic pregnant patients (R^2^ 0.27, *p* value = 0.002; R^2^ 0.31, *p* value = 0.001; R^2^ 0.35, *p* value = 0.001, respectively). ANOVA revealed a statistically significant difference in fibrinogen concentration between all three patients groups when assayed using the Clauss method (*p* value < 0.001 for all the comparisons), but no statistically significant difference between the two patients groups of pregnant women when using the FLEV method.

**Conclusions:**

The FLEV method does not provide a valid alternative to the Clauss method due to the problem of fibrinogen overestimation, and for this reason it should not be recommended for the evaluation of patients with an increased risk of hypofibrinogenemia.

## Background

Hemostasis is the dynamic equilibrium between coagulation and fibrinolysis. During pregnancy, the balance shifts to a hypercoagulative state that becomes more pronounced toward the end of the third trimester, returning to normality approximately 4 to 5 weeks after delivery. Hypercoagulability results from an increase in plasma concentrations of coagulation factors VII, VIII, X, XII, von Willebrand factor (vWF), and fibrinogen (which can reach 6 g/L by the end of pregnancy) [1]. Gestational thrombocytopenia may also occur during the third trimester with platelet counts dropping by approximately 10% with respect to baseline [2]. Fibrinolysis is also markedly depressed during a normal pregnancy [2]. It is important to highlight that the coagulation changes occurring during postpartum hemorrhage (PPH) differ from those of polytraumatized or postsurgical patients because of the underlying cause of obstetric bleeding [3].

Uterine atony, genital tract trauma, and surgical trauma are not always associated with development of coagulopathy, although they may cause significant blood loss. However, uncontrolled bleeding in this context may evolve into a late coagulopathy [4–7]. In contrast, placental abruption (even with minimal blood loss) and abnormal placentations may be associated with rapidly evolving coagulopathy characterized by the consumption of coagulation factors. Placental abruption and amniotic fluid embolism are the main causes of the onset of disseminated intravascular coagulopathy (DIC) [1, 8].

During PPH, fibrinogen is of fundamental importance, and a blood level of fibrinogen less than 2 g/L (200 mg/dL) is a positive predictive value for severe PPH and the need for angiographic invasive procedures [9, 10], higher blood and plasma transfusion, and a longer stay in the intensive care unit [11–15]. A reliable and rapid method for determining fibrinogenemia is therefore essential in order to be able to intervene quickly. Functional fibrinogen (FLEV) assessment by TEG [16] and the gold standard laboratory Clauss method are the two most widespread methods for assaying circulating fibrinogen levels.

FLEV, as a point-of-care (POC) test, has the advantage of providing results more rapidly, however, concerns have been raised about the accuracy of FLEV measurement in patients with a hemorrhage in progress, although the obstetric context has never been specifically analyzed until now. Several studies have reported a good correlation between functional fibrinogen measured by TEG (FLEV) and laboratory- diagnosed fibrinogenemia as assessed using the Clauss method, whereas other studies have shown TEG to overestimate actual levels [17–19]. Specifically, TEG estimates the functional fibrinogen level (FLEV), by extrapolation from the MA (maximal amplitude) fibrinogen value. The MA value of a platelet-free plasma clot is proportionate to the functional fibrinogen concentration. Analytical software is able to calculate the functional fibrinogen level (MAFF or FLEV) by transformation of the MA value. The gold standard method, however, is the Clauss assay that needs to be carried out in a clinical laboratory. For its execution, a standard curve is created by determining the thrombin time for different plasma dilutions with a known fibrinogen concentration. In brief, a citrated whole blood sample is taken from a patient, centrifuged, and the plasma portion stored. The plasma is then diluted 1:10 and the thrombin time calculated. The measured thrombin time is then placed on the standard curve and the fibrinogen concentration extrapolated.

The aim of the present study was to evaluate the concordance between the two most widely used methods of fibrinogen measurement – TEG and the Clauss method – in i) healthy volunteers, ii) non pathological pregnant patients, and iii) pregnant patients who developed PPH.

## Methods

### Materials and methods

This prospective observational study was conducted at the University Hospital of Udine and approved by the local Ethics Committee (prot. N. 17534). Inclusion criteria were: healthy volunteer women of childbearing age (“healthy volunteers”), non-pathological pregnant women at term (“non-pathological pregnant patients”) and pregnant hemorrhagic patients (“hemorrhagic pregnant patients”) subjected to elective or urgent caesarean section (CS), with blood loss exceeding 1000 mL. Exclusion criteria were age < 18 years, a history of coagulopathy, and treatment with contraceptives, anticoagulants, or antiplatelet agents.

For each patient, the following preoperative data were collected: age, gestational age, and reason for cesarean section. The intraoperative data collected consisted of the following blood levels/values: hemoglobin (Hb), hematocrit (HCT), red blood cells, platelets, PT, aPTT, INR, D-Dimer, Antithrombin (AT), Clauss fibrinogen, thrombolelastographic parameters (R, K, Angle ɑ, MA, CI, Ly30), FLEV, and the volume of blood loss. If blood loss exceeded 1000 mL, the patients were designated to the “hemorrhagic pregnant patients” group. For healthy volunteers, we recorded hemoglobin (Hb), hematocrit (HCT), red blood cells, platelets, PT, aPTT, INR, D-Dimer, Antithrombin (AT), Clauss fibrinogen, TEG parameters, and FLEV. In the operating room, all patients were monitored for heart rate (HR), noninvasive blood pressure (NIBP), peripheral arterial saturation (SpO_2_), and EtCO_2_ with in-out gas analysis. Regional or general anesthesia was performed according to internal protocols. Blood samples for thromboelastographic examination were collected into a blood tube containing citrate (0.13 M) and analyzed using a TEG® 5000 Thrombelastograph® Hemostasis Analyzer (Haemoscope Corporation, Niles, IL, USA). This point-of-care instrument was subjected to a daily quality control protocol (e-test, bubble test and level 1 and 2 controls), and the manufacturer’s instructions were always followed. The staff performing the tests had undergone comprehensive training. In our Institute, staff are also subjected to periodic evaluations to check their ability to perform the tests. The TEG FLEV calculation was performed by the TEG® system’s internal software (Haemoscope Corporation, Niles, IL, USA). A blood volume equal to 360 μL was taken from the sampling tube and placed, using a special pipette, in a preheated cuvette at 37 °C containing 20 μL calcium for TEG parameter analysis.

To perform the functional fibrinogen (FF) assay (Clauss method), 0.5 mL of citrated blood was added to the designated FF vial containing abciximab (a monoclonal antibody that inhibits platelet aggregation), tissue factor (a glycoprotein necessary for the formation of thrombin), sodium azide (the sodium salt of hydrogen azide – a preservative of biological fluids), and tris buffer (buffer salt solution for pH management) and gently mixed. A 340 μL aliquot was transferred from the FF vial to a 37 °C preheated TEG cuvette preloaded with 20 μL 0.2 M CaCl_2_. The samples were analyzed within 30 min of sampling, and the thromboelastographic trace was generated and analyzed within 90 min. The samples for both thromboelastography and the Clauss assay were collected simultaneously. Blood samples for hemoglobin, hematocrit, red blood cell and platelets evaluation were collected into tubes containing ethylenediaminetetraacetic acid (EDTA); samples for hemogens and fibrinogen analysis were collected into tubes containing citrate 0.13 M.

### Statistical analysis

Considering a linear correlation of 0.5 (for an alpha value of 5% and a statistical power of 90%), we calculated a minimal sample size of 32 patients for each group. Descriptive statistics were calculated for the main study variables. For the comparison of qualitative variables, we considered frequencies and percentages; for quantitative variables, we considered means and standard deviations (SD). The Bland-Altman plot was used to evaluate the level of agreement between the results of the Clauss method and FLEV for each group [20]. The correlation between the two measures of fibrinogen and between platelets, hemoglobin, and the TEG parameters (maximum amplitude [MA] and the alpha angle) was studied using the Spearman correlation coefficient calculated for each group. The relationship between the two methods of fibrinogen determination was analyzed for each group by linear regression analysis. A *p* value ≤0.05 was considered significant. A multiple comparison between groups for both methods of fibrinogen determination was made using ANOVA. A multiplicity adjustment was obtained using the Westfall test. All statistical analyses were performed using R-Cran ver. 3.4.2 language and environment for statistical computing (R Core Team; R Foundation for Statistical Computing, Vienna, Austria, http://www.R-project.org).

## Results

Between October 2016 and June 2017, 103 participants were enrolled onto the study. Two patients were excluded for a distorted TEG trace due to technical problems and a further 3 due to delays in the samples arriving in the clinical laboratory. The final number of participants was 98: 32 healthy volunteers, 34 pregnant patients at full-term, and 32 pregnant patients with hemorrhage. No participants were found to have coagulation abnormalities or were being treated with antiplatelet or anticoagulant therapies. The characteristics of the studied population are shown in Table 1. The studied variables are shown in Table 2.

**Table 1:** Characteristics of the studied population. Values are expressed as median and, in brackets, the interquartile values

	Healthy volunteers (*n* = 32)	Non-pathological Pregnant Pts (*n* = 34)	Hemorrhagic Pregnant Pts (*n* = 32)
Age, years	37 (26–40)	34.5 (31–38)	37.5 (32–40)
Height, cm	167 (163–170.3)	165 (160–168)	167 (163–170.3)
Weight, Kg	75.5 (69.5–87.3)	70.5 (64–80.8)	75.5 (69.5–87.3)
BMI	26.9 (24.2–29.3)	27.2 (25–28.2)	26.9 (24.2–29.3)
Gestational age, weeks + days	36 + 5 (35–37 + 5)	39 (38 + 3–39)	36 + 3 (35 + 4–37 + 2)

**Table 2:** Studied variables. Values are expressed as median and, in brackets, the interquartile values

	Healthy volunteers (*n* = 32)	Non-pathological Pregnant Pts (*n* = 34)	Hemorrhagic Pregnant Pts (*n* = 32)
FBN Clauss, mg/dL	257.4 (241.4–294.1)	489.8 (437.5–524)	365.5 (307.7–416.5)
FLEV, mg/dL	386.9 (351.8–448.5)	525.6 (490.5–589)	521.9 (483.6–565.7)
MA TEG, mm	55.3 (50.8–60.3)	70.3 (68.3–73)	71.4 (67.1–74.6)
MA FLEV, mm	21.2 (19.3–24.6)	28.8 (26.9–32.3)	28.6 (26.5–31)
PLT, 10^3/μL	237.4 (202.6–263.1)	183.5 (160.9–218.1)	161.7 (132.3–181.5)
Hb, g/dL	13.1 (12.4–13.6)	11.8 (11.4–12.4)	10 (9.2–10.5)

**Fig. 1:**
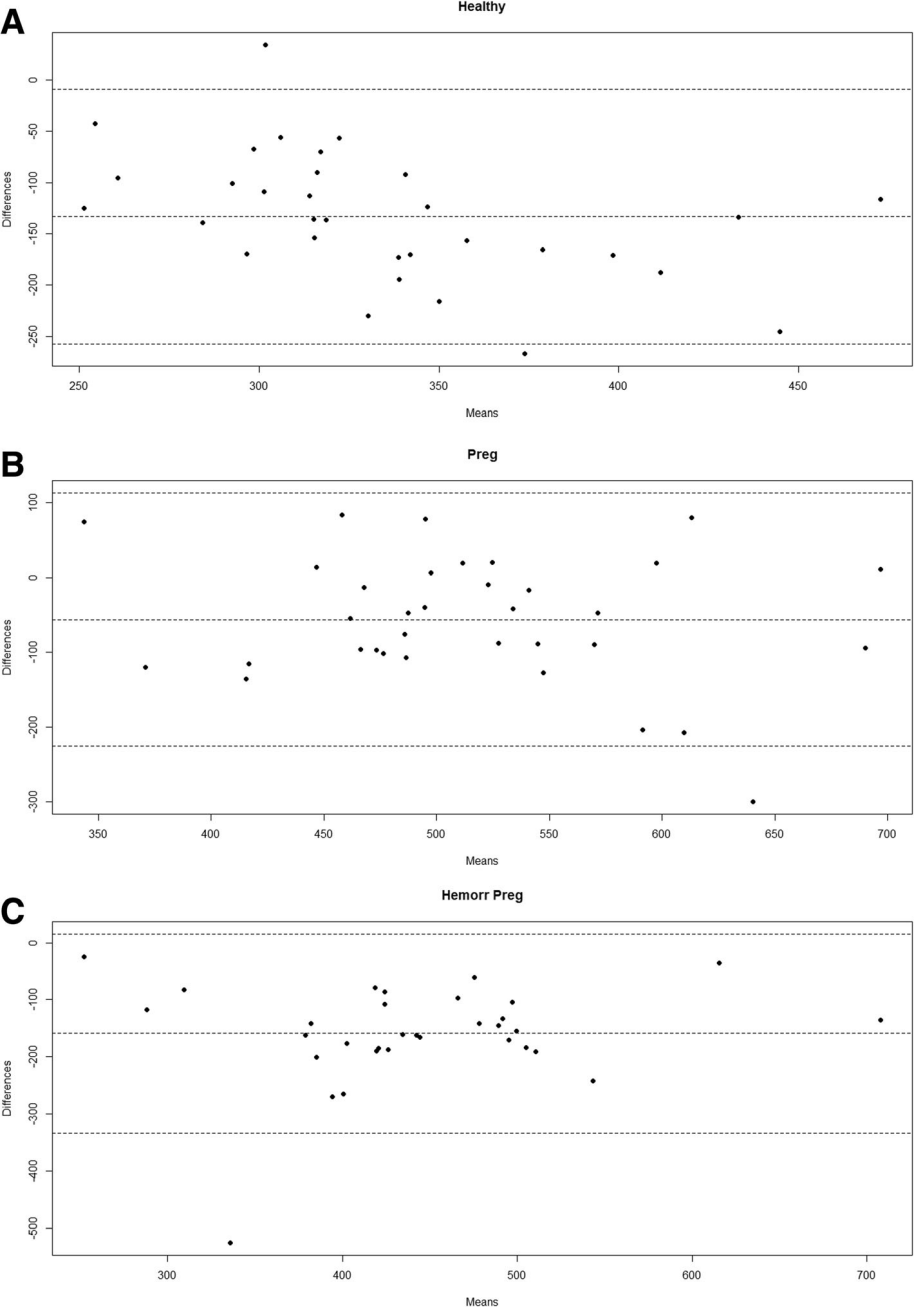
Bland-Altman charts for each group considered (healthy volunteers, ie "Healthy", non pathological pregnant patients, ie "Preg", and hemorrhagic pregnant patients, ie "Hemorr Preg"). On the y-axis the differences are set, the measured fibrinogen values are placed on the x-axis

**Fig. 2:**
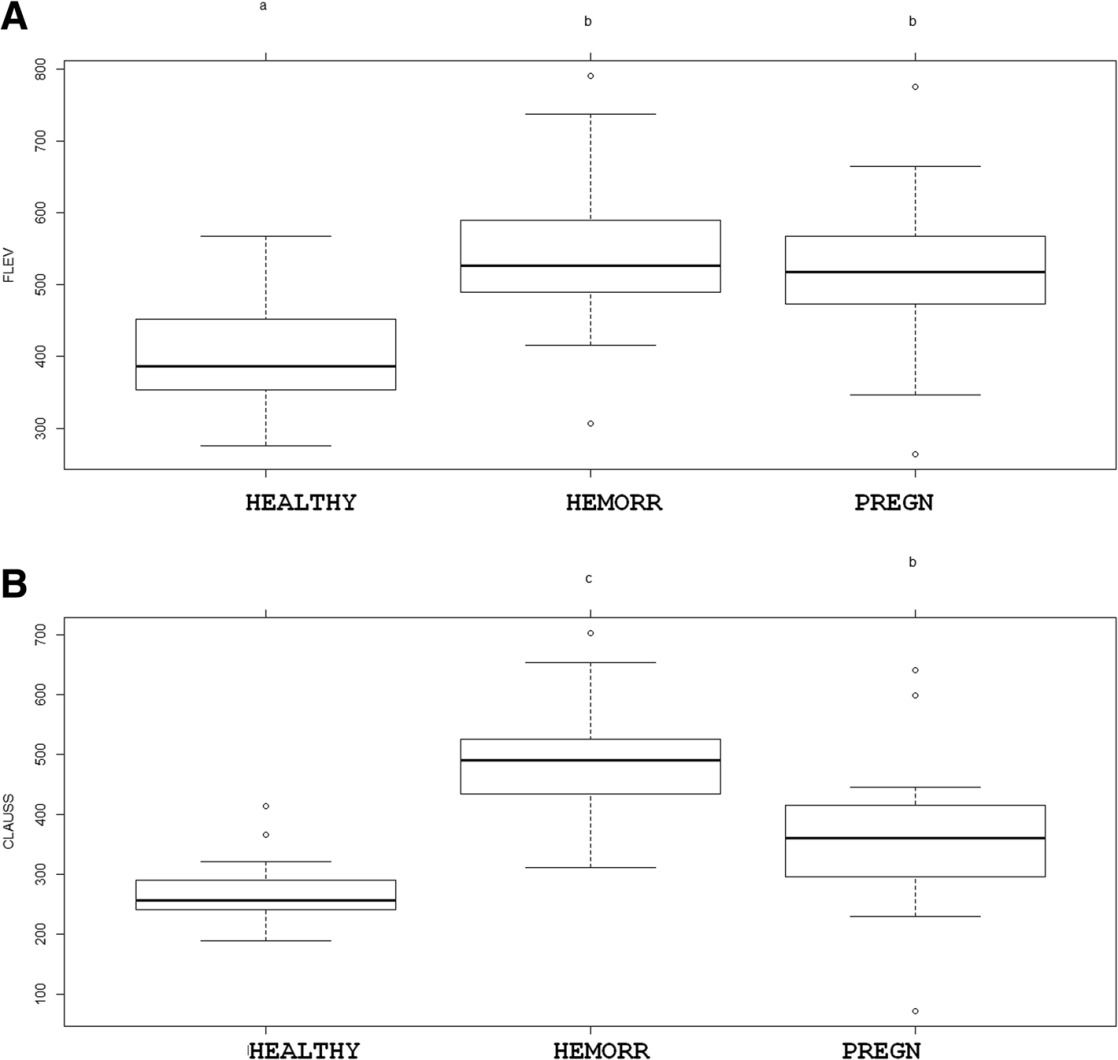
Box plots for the method of determining the fibrinogen of Clauss (**a**) and FLEV (**b**). Fibrinogen values are placed on the y axis. On the x axis are placed the three different samples analyzed (healthy volunteers, hemorrhagic pregnant patients and non pathological pregnant patients). The letters above the graphs refer to different clusters of significance: a different letter corresponds to a statistically significant difference between the groups

The Bland-Altman plots showed fairly good correlation between the two measures, but the FLEV measurements consistently were consistently higher than those obtained quantitatively by the Clauss method: bias was − 133.36 mg/dL for healthy volunteers (95% IC: − 257.84; − 8.88. Critical difference: 124.48) (Fig. 1a); − 56.30 mg/dL for healthy pregnant patients (95% IC: − 225.53; 112.93. Critical difference: 169.23) (Fig. 1b); and − 159.05 mg/dL for hemorrhagic pregnant patients (95%IC: − 333.24; 15.148. Critical difference: 174.19) Fig. 1c. In 3 of the 32 cases of pregnant women with postpartum hemorrhage, clinical treatment of fibrinogenemia was only initiated once the laboratory results had been obtained that revealed the overestimation of FLEV by TEG (that had provided an incorrect estimate above 250 mg/dL). The Spearman correlation between FLEV and Clauss was 0.39 (*p* = 0.027) in healthy volunteers, 0.54 (*p* = 0.001) in the pregnant term patients, and 0.57 (*p* = 0.001) in the hemorrhagic pregnant patients. Regression analyses detected a linear correlation between FLEV and Clauss for healthy volunteers, healthy pregnant patients, and hemorrhagic pregnant patients (R^2^ 0.27, *p* value = 0.002; R^2^ 0.31, *p* value = 0.001; R^2^ 0.35, *p* value = 0.001, respectively). ANOVA analysis demonstrated statistically significant differences in fibrinogen assayed using the Clauss method between the three groups of patients (*p* value < 0.001 for all the comparisons) (Fig. 2b). On the contrary, no statistically significant difference was present between the two groups of pregnant patients when the FLEV method was used (*p* value < 0.001 for the comparisons between healthy volunteers and pregnant patients; *p* value = 0.186 for the comparison between healthy and hemorrhagic pregnant patients) (Fig. 2a).

## Discussion

The main finding of the current work is that fibrinogen estimation by FLEV in pregnant term women and hemorrhagic pregnant patients does not correlate closely enough with the levels obtained via the quantitative Clauss assay.

The FLEV methodology was developed in order to obtain precise measures of fibrinogen as fast as possible, i.e., at the bedside. In particular, its use would bring particular benefit to patients with fibrinogen levels lower than 250 mg/dL, so to permit its rapid correction in cases of acute bleeding. As a matter of fact, fibrinogen measurements have been incorporated into the latest transfusion algorithms for patients undergoing cardiac surgery, polytrauma patients, and the management of pregnant patients developing postpartum hemorrhage, for whom early correction is essential for levels lower than 250 mg/dL. Indeed, fibrinogenemia less than 250 mg/dL has been identified as an early marker of progression to larger volume and more prolonged hemorrhage, higher rates of red blood cell and plasma transfusion, invasive angiographic procedures, and prolonged hospital stays.

The management protocol of massive hemorrhage bleeding highlights the importance of fibrinogenemia correction, which, in addition to laboratory tests, recommends the performance of viscoelastic methods, i.e., rotational thromboelastometry (ROTEM) or thromboelastography (TEG) when available. TEG seems to be a promising application in that it is a rapid test that does not require highly specialized personnel and results are available in only 15–20 min. By contrast, the time required to complete a Clauss assay is two- or even three-fold that for TEG, requiring 40 to 60 min.

Regarding the two techniques, ROTEM has showed better predictive accuracy than TEG in cardiac surgery and trauma patients [19, 21, 22]. Whereas in pregnant women and liver transplantation patients, great variability was revealed in the results for MA-FF vs. Clauss and FIBTEM (which is a point-of-care method that eliminates the platelet contribution of clot formation by inhibiting the platelets irreversibly with cytochalasin D) vs. Clauss [23].

Our results diverge from those of Harr et al. [19], who found a close correlation between FLEV and fibrinogen assayed using the Clauss method in 68 polytrauma patients (R^2^ = 0.80). Moreover, Pruller et al. [24] obtained a fairly good correlation between FLEV and Clauss (R^2^ = 0.54) in surgical patients. However, conflicting results have been reported in the literature, depending on the populations studied; Agarwal et al. [25], for example, found a weak correlation in cardiac surgical patients (R^2^ = 0.11).

Our results show that the two methods are not interchangeable because a systematic overestimation obtained by TEG compared with the Clauss method. In agreement with our data, Katz et al. [26], in 56 parturients, demonstrated a propensity for the point-of-care method (FLEV) to overestimate compared with the laboratory approach (Clauss), especially when the fibrinogen levels increased above 500 mg/dL (SD 52.8 mg/dL). Agren et al. [18] obtained similar results, with an overestimation obtained by FLEV of about 100 mg/dL compared with the Clauss method. The degree of overestimation detected in the present study was even greater, especially in pregnant patients with hemorrhage for whom greater accuracy is essential – especially since the comparison of fibrinogen levels between healthy pregnant and pregnant patients with hemorrhage revealed no statistical difference for FLEV, whereas the difference did achieve statistical significance with the Clauss method, which could distinguish the two populations based on fibrinogen levels. Once again, we must highlight the possibility that an overestimation of fibrinogen level by FLEV could cause a delay in treatment in clinical practice.

What underlies the difference between the two tests? First of all, Clauss is a quantitative method, whereas FLEV is qualitative. Second, FLEV measures the fibrinogen in whole blood, whereas the Clauss method uses plasma [27]. Third, the non-concordance between FLEV and the Clauss method is probably due to the impossibility of obtaining a complete inhibition of platelets in whole blood samples. The lyophilized tissue factor and the abciximab that binds to glycoprotein IIb/IIIa receptors inhibit platelet aggregation and exclude the contribution of platelets to clot strength. However, Lang et al. demonstrated [28] that abciximab does not inactivate the glycoproteins completely. Furthermore, when the number of platelets increases, a smaller percentage is inhibited and the inaccuracy of the FLEV value increases. Fluid management during anesthesia may also play a role [29, 30]. Last, but not least, hematocrit and activated factor XIII could have an impact on clot firmness and affect the correlation [31, 32]. As discussed above, we recommend continuation of the Clauss laboratory reference method; hospital staff should endeavor to shorten the delivery time of blood samples to the laboratory and to speed up subsequent processing times through, for example, utilization of a priority channel.

### Limitations

The FLEV and the Clauss values are expressed as analytical variables. We conducted frequent quality controls; double assays of analyzed samples were often performed to minimize the pre-analytical error, and the values set as the laboratory reference range are obtained from the average of a large pool of healthy volunteers. The major limitation of the FLEV method is the incomplete inhibition of platelets with the current reagent.

## Conclusions

At present, FLEV should not be considered an interchangeable alternative to the Clauss method, especially when dealing with pregnant term women and hemorrhagic pregnant patients because it overestimates the fibrinogen level in the blood. As such, it should not be used in the treatment of hemorrhagic patients with hypofibrinogenemia. Therefore, at present, it is reasonable to use the Clauss method by constructing a specific protocol with an emergency channel to shorten sample analysis times and guarantee the timely correction of hypofibrinogenemia.

## Data Availability

Data is available if requested.

## Abbreviations

ANOVA: Analysis of variance
aPTT: Activated partial thromboplastin time ratio
AT: Antithrombin
CS: Caesarean section
DIC: Disseminated intravascular coagulopathy
EDTA: Ethylenediaminetetraacetic acid
EtCO2: End tidal carbon dioxide
FF: Functional fibrinogen
FLEV: Functional fibrinogen level
Hb: Hemoglobin
HCT: Hematocrit
HR: Heart rate
INR: International normalized ratio
NIBP: Non-invasive blood pressure
PPH: Postpartum hemorrhage
PT: Prothrombin time
ROTEM: Rotational thromboelastometry
SD: Standard deviation
SpO2: Peripheral arterial saturation
TEG: Thromboelastography
vWF: Von Willebrand factor
